# Cannabis and Illicit Drug Use During Neurodevelopment and the Associated Structural, Functional and Cognitive Outcomes: Protocol for a Systematic Review

**DOI:** 10.2196/18349

**Published:** 2020-07-27

**Authors:** Jennifer Debenham, Nicola Newton, Louise Birrell, Murat Yücel, Briana Lees, Katrina Champion

**Affiliations:** 1 The Matilda Centre for Research into Mental Health and Substance Use The University of Sydney Sydney Australia; 2 Brain & Mental Health Laboratory Monash Institute of Cognitive and Clinical Neurosciences Monash University Melbourne Australia

**Keywords:** cannabis, illicit drugs, neurodevelopment, longitudinal review, protocol, neurology, review

## Abstract

**Background:**

High rates of cannabis and illicit drug use are experienced by young people during the final stages of neurodevelopment (aged 15-24 years), a period characterized by high neuroplasticity. Frequent drug use during this time may interfere with neurophysiological and neuropsychological development pathways, potentially leading to ongoing unfavorable neuroadaptations. The dose-response relationship between illicit drug use, exposure, and individual neurodevelopmental variation is unknown but salient with global shifts in the legal landscape and increasingly liberal attitudes and perceptions of the harm caused by cannabis and illicit drugs.

**Objective:**

This systematic review aims to synthesize longitudinal studies that investigate the effects of illicit drug use on structural, functional, and cognitive brain domains in individuals under the neural age of adulthood (25 years). This protocol outlines prospective methods that will facilitate an exhaustive review of the literature exploring pre- and post-drug use brain abnormalities arising during neurodevelopment.

**Methods:**

Five electronic databases (Medline, Embase, PsycINFO, ProQuest Central, and Web of Science) will be systematically searched between 1990 and 2019. The search terms will be a combination of MeSH (Medical Subject Headings), with keywords adapted to each database. Study reporting will follow the Preferred Reporting Items for Systematic Reviews and Meta-Analyses (PRISMA) guidelines, and if relevant, study quality will be assessed using the Grades of Recommendation, Assessment, Development, and Evaluation (GRADE) approach. Eligible studies are those that sampled youth exposed to cannabis or illicit drugs and employed neurophysiological or neuropsychological assessment techniques. Studies will be excluded if participants had been clinically diagnosed with any psychiatric, neurological, or pharmacological condition.

**Results:**

This is an ongoing review. As of February 2020, papers are in full-text screening, with results predicted to be complete by July 2020.

**Conclusions:**

Integrating data collected on the three brain domains will enable an assessment of the links between structural, functional, and cognitive brain health across individuals and may support the early detection and prevention of neurodevelopmental harm.

**Trial Registration:**

PROSPERO CRD42020151442; https://www.crd.york.ac.uk/prospero/display_record.php?RecordID=151442

**International Registered Report Identifier (IRRID):**

PRR1-10.2196/18349

## Introduction

Illicit drug use and associated harms are highest among young people aged 15-24 years [1]. Globally, the onset of illicit drug use spikes around 15 years of age, a stage of high neuroplasticity, and rates climb throughout emerging adulthood (18-24 years) before dropping at approximately 25 years—after neuromaturation [2,3]. It comes as no surprise that illicit drug use during neurodevelopment is widespread; a quarter (24%) of adolescents (12-17 years) and over half (57%) of emerging adults report illicit drug use in the United States [4]. Moreover, from adolescence to emerging adulthood, lifetime methylenedioxymethamphetamine (MDMA; ecstasy) use surges from 0.8% up to 10.5% and cocaine use rises from 0.7% to 11.4% [4]. Although single-time drug use carries a small increase in morbidity and mortality, it may be the patterns of use and stage of neurodevelopment, that most influences the risk of neuronal aberrations and dependence [5]. Cannabis is the most commonly consumed illicit drug worldwide, with 5.6% of the world’s adolescent population reporting recent use, nearly one fifth (18%) of Europeans (15-24 years) reporting past-year use [6], and 10% of young Canadians reporting daily or almost daily use [7]. In Australia, around 41% of young people (14-24 years) have used an illicit drug in the past year, representing 90% of all national illicit drug users [8]. Despite risky patterns of use and the potential for ongoing harm, there is limited research investigating the ongoing impact of illicit drug use on the developing brain.

Adolescence and emerging adulthood represent a period of protracted neurobiological development marked by structural and functional remodeling, contributing to improved cognitive performance [9,10]. Structural integration occurs throughout the brain into early adulthood, with processes such as synaptic pruning (removal of unneeded neural connections), myelination (insulation and strengthening of neural messaging), and synaptogenesis (new neural connections), occurring predominantly in the prefrontal cortex [11]. These structural advances provide the underpinning of encephalization, which is the age-related transfer of function from the primitive, autonomic systems such as the hindbrain (pons, cerebellum, and medulla oblongata) and subcortical midbrain (limbic system), to the sophisticated cognitive systems of the forebrain (cerebral cortex, including the prefrontal cortex) [12,13]. Structural and functional maturation in cortical regions of the brain, specifically in the prefrontal cortex, precipitate improved cognition functioning [14], however, occur over a nonlinear trajectory. For example, neural projections between the prefrontal cortex and subcortical limbic system oscillate between regressive and progressive changes. Although development across structural, functional, and cognitive domains are inextricably linked, they are asynchronous, so one does not indicate the presence of another. Therefore, interpreting single brain outcomes during neurodevelopment is limited, particularly if assessed during a single data instance. Triangulating neurophysiological and neuropsychological studies may help to account for different trajectories of neurodevelopment and establish a more wholistic view of brain health.

The neurotoxic impact of illicit drugs on the developing brain has been studied extensively; however, a clear relationship between exposure (drug class[es] and patterns of use) and neurological sequelae is yet to be established. Sustained use of most drugs, such as cannabis inhalants, opiates, psychostimulants, and ecstasy, tends to be associated with executive dysfunction such as declines in working memory, verbal fluency, learning, and attention [15-18] and structural and functional abnormalities in the frontal cortex and limbic system [19-22]. However, the precise and quantifiable impact of different drug classes is less clear. A recent review of neuroimaging publications involving adolescent drug use showed that most focus on alcohol, only 45% focus on cannabis, less than 2% assess ecstasy/meth and inhalants, and 7% address polydrug use [23]. Untangling the impact of illicit substance use, including the difference between regular low dose use and infrequent high dose use, is challenging in part due to the limited investigation in this area [24,25]. Studies measuring the brain’s capacity to recover after abstinence are mixed [26,27], with some confirming cognitive deficits are present in the weeks after drug use [28,29], others claiming full recovery [30], and others showing persistent structural and functional changes months and years after abstinence [29,31]. Longitudinal study designs are required to discern the impact of complex patterns of illicit drug use and determine which classes carry additional risk of ongoing harm.

The age of drug use onset and corresponding stage of brain development may mediate neurophysiological and neuropsychological vulnerability to harm [32,33]. Many studies have shown a difference in harm between adolescents and adults [25], and some researchers hypothesize the brain is more protected against harm after 16 or 17 years of age [34,35]. Adding to the complexity are sex-based neurobiological factors that may subject females to higher risk than males [36-38]. Unfortunately, sex-based differences are not well understood, in part due to the underrepresentation of females in neuroimaging studies. Pressingly, the gender gap between male and female use may be closing [39-41] and could be accompanied by an increase in the number of young females presenting with neurodevelopmental aberrations. Longitudinal studies that help to untangle the relationship between pre-existing neural differences and trajectories of neurodevelopment will help elucidate individual vulnerability to harm.

Several narrative reviews have synthesized the evidence of the long-term impact of illicit drug use on the developing brain [42,43]; however, there are limited systematic reviews that can provide a complete view of the evidence. The systematic reviews are highly skewed towards cannabis use [44-46] and tend to provide insight into a single brain outcome [47]. Very few reviews harness structural, functional, and cognitive data [23,48-50], and only one incorporates findings on more than one drug [51]. Most importantly, very few reviews assess the longitudinal harms of drugs [25,52,53] and instead rely on cross-sectional data, which provide a limited assessment of change over neurodevelopment. Of these reviews, one did not include neuroimaging data [54], and the remaining three included only one brain domain [25,52,53]. To our knowledge, there has been no systematic review of longitudinal studies measuring the long-term impacts of a broad range of common illicit drugs, with findings encompassing both neurobiological and neuropsychological outcomes. To understand the neurological health burden of illicit drugs for young people and to inform policy and future prevention programs, a systematic review of the longitudinal studies assessing the impact of illicit drug use on structural, functional, and cognitive brain domains in young people will be conducted. Specifically, the main objectives of this review include:

1. Investigate pre-existing neurodevelopmental risk factors associated with increased cannabis and illicit drug use and related harm for young people (<25 years).
2. Assess the residual, dose-dependent effects of cannabis and illicit drugs on the function, structure, and cognitive profile of the developing brain (<25 years).
3. Determine to what extent cannabis and illicit drug use during neurodevelopment (<25 years) predict sustained structural, functional, and cognitive brain changes.

## Methods

This systematic review has been registered with the International Prospective Register of Systematic Reviews (PROSPERO; CRD42020151442) and was written per the Preferred Reporting Items for Systematic Review (PRISMA) guidelines [55] as provided (see Multimedia Appendix 1).

### Population Characteristics

Studies that target young people aged 10-25 years who have any lifetime exposure to cannabis or another illicit drug will be included. We will exclude studies in which the average age of participants is older than 25years.

### Eligibility Criteria

The proposed systematic review will include published studies that focus on young people aged 10-25 years who have used cannabis or an illicit drug; report on empirical, longitudinal data, where the same measurement has been employed over multiple time points; and where the measurements include test batteries assessing structural, functional or neuropsychological brain domains. Studies will include a sample with exposure to cannabis, illicit drugs, or both, where exposure involves at least single time use. Studies must compare participants who meet the criteria for cannabis or illicit drug exposure with a comparison group with less exposure. Eligible studies published after January 1990 in the English language will be included.

Participants who have been clinically diagnosed with psychiatric or neurological conditions will be excluded from the review to prevent confounding results. As this review focuses on brain changes over time, we will exclude cross-sectional studies.

### Search Strategy

Aided by a research librarian, five electronic databases will be systematically searched: Medline (Ovid), Embase (Elsevier), PsycINFO (OvidSP), ProQuest Central, and Web of Science, using Medical Subject Headings (MeSH). Search terms will be developed individually for each database, and specific terms for each search group are defined in the additional search strategy file (see Multimedia Appendix 2 for strategy and Multimedia Appendix 3 for an example search of Medline). The search will be limited to peer-reviewed studies of human subjects published in the English language, between 1990 and 2019, given the improvement in brain scanning techniques during this time. Researchers will manually review the reference lists of eligible papers to detect further relevant papers and will cross-reference other recent systematic reviews to discover additional studies. All papers identified in the search strategy will be exported into a bibliographic database for deduplication and screening and uploaded to the Covidence online software program for screening.

### Data Extraction and Screening

All titles and abstracts from the returned searches will be screened by one reviewer based on the eligibility criteria, and a random sample of 25% will be screened again by a second reviewer, with any disagreement resolved by a consensus. Similarly, full-text copies will be screened twice according to the eligibility criteria by two independent reviewers with any disagreement resolved by a consensus. Data extraction will be supported by an extraction template, which will first be piloted to ensure it adequately captures trial data. The following data will be extracted from all included full-text articles:

- Study information (author, year, title, location);
- Study characteristics (study design, imaging/test technique);
- Sample characteristics (sample size, gender and age distribution);
- Drug exposure characteristics (drug type/s, age of initiation, route of administration, frequency, quantity, abstinence period, and assessment method);
- Data characteristics (exclusion criteria, number of measurement occasions, brain region);
- The statistical approach used to investigate change over time; and
- Key results.

Where necessary, the corresponding author of the included studies will be contacted via email to obtain any relevant data not presented in the published paper. In the case of study attrition bias, authors will be contacted to request additional data to be incorporated into the review.

### Outcomes

The primary outcomes of interest will be the residual and long-term differences in neurobiological (structural and functional) and neuropsychological (cognitive) brain domains between the active and control group. The neuroanatomical development trajectories of particular interest include global and local gray matter measures (volume, density, and thickness); integrity of white matter microstructure and fiber connectivity (including directional organization, myelination, axonal packing) [56]; and receptor distribution. Global and local function, as understood through cortical activation, will be measured by blood flow and blood oxygenation level-dependent oxygen consumption [57]. Finally, cognitive performance, will be measured through neuropsychological tasks assessing attention and concentration, decision-making and risk-taking, inhibition and impulsivity, episodic and working memory, verbal fluency, planning, IQ and general executive functioning. Where possible, outcomes will be deconstructed into male and female results.

### Strategy for Data Synthesis

We anticipate a high degree of heterogeneity among study design and participant characteristics, and will, therefore, conduct a narrative synthesis on all available data. If it is appropriate to combine studies, meta-analysis will be conducted using Review Manager software (Cochrane). Synthesis of the included study data will be structured according to technique type, and differences between the control and exposed groups will be compared. Pre-existing cognitive, structural, and functional features will be accounted for through standardization. If possible, a subanalysis of gender-based differences will be conducted.

### Risk of Bias and Quality Assessment

Reviewers will independently assess the risk of bias by adapting Cochrane Collaboration’s tool for assessing the risk of bias in trials [58]. The tool will be used to assess the extent to which biases may impact study results, such as selection bias, attrition bias, reporting bias, performance bias, and detection bias. Other relevant biases will be considered where appropriate. A third reviewer will resolve any discrepancies that may arise. Reviewers will assign scores to the six domains, and the total risk of bias will be provided for each study, with higher scores indicating a lower risk of bias.

The overall quality of the body of evidence will be determined using the Grades of Recommendation, Assessment, Development, and Evaluation approach [59]. Study quality will be graded as high (further research is unlikely to change our confidence in the effect), moderate (further research is likely to have an impact on our confidence in the effect and may change the estimate), low (further research is likely to have an impact on our confidence in the effect), and very low (uncertain about the effect estimate). The included studies will start with a high-quality rating and move downwards based on scoring, and observational studies will begin on a low-quality rating and move upwards.

## Results

This paper describes an ongoing review. As of February 2020, papers are in full-text screening, with results predicted to be complete by July 2020.

## Discussion

Accumulating research assessing the impact of illicit drug use on the developing brain underscores the need for a systematic review to assist clinicians, educators, and public health advisors in evidence-based practice. The significant consequences of illicit drug use, such as an increase in the likelihood of developing a drug use disorder or a persisting mental deficit, may be preventable [37,60]. The shifting legal landscape and subsequent liberalization of attitudes and perception of the harm of drugs make quantifying their precise neurological impact critically important. Synthesizing a current view of the evidence of illicit drug use and brain development may support the early detection and prevention of neurodevelopmental harm.

## Appendix

Multimedia Appendix 1 PRISMA-P (Preferred Reporting Items for Systematic Review and Meta-Analysis Protocols) checklist.

Multimedia Appendix 2 Example MeSH (Medical Subject Headings) from search strategy.

Multimedia Appendix 3 Example of search strategy in Medline/Ovid.

## Abbreviations

GRADE: Grades of Recommendation, Assessment, Development, and Evaluation
MDMA: methylenedioxymethamphetamine
MeSH: Medical Subject Headings
PRISMA: Preferred Reporting Items for Systematic Reviews and Meta-Analyses
PROSPERO: International Prospective Register of Systematic Reviews
