# Supplementary material for: Cannabis and Illicit Drug Use During Neurodevelopment and the Associated Structural, Functional and Cognitive Outcomes: Protocol for a Systematic Review
Source: JMIR Res Protoc. 2020 Jul 27;9(7):e18349. doi: 10.2196/18349 (PMC7418018; doi:10.2196/18349)
Supplement: Multimedia Appendix 2 [file resprot_v9i7e18349_app2.pdf]

Example medical subject headings (MeSH) from the Search Strategy

**Exposure**	**Participants**	**Outcome**
MDMA	Adolescen*	Neuroimag*
Ecstasy	teenage*	neuropsychological test
Cannabis	young people	neuroscience
Marijuana	youth	PET scan
Cocaine	emerging adult	brain imag*
Methamphetamine	young adult	spectroscop*
amphetamine	college student*	magnetic resonance imag*
crystal meth	*m.p.*	fMRI
illicit drug*		sMRI
illicit substance*		magnetic resonance spectroscopy
street drug*		electroencephalogram
m.p.		diffusion tensor imag*
		neuropsychological
		cogniti*
		verbal working memory
		episodic memory
		visuospatial working memory
		verbal fluency test
		executive function*
		structural imag*
		functional imag*
		m.p.
