# Supplementary material for: Cannabis and Illicit Drug Use During Neurodevelopment and the Associated Structural, Functional and Cognitive Outcomes: Protocol for a Systematic Review
Source: JMIR Res Protoc. 2020 Jul 27;9(7):e18349. doi: 10.2196/18349 (PMC7418018; doi:10.2196/18349)
Supplement: Multimedia Appendix 3 [file resprot_v9i7e18349_app3.pdf]

Example of search strategy in MEDLINE/Ovid database.

Search Strategy: September 19, 2019

#	Searches	Results
1	MDMA.mp. or exp midomafetamine/	4069
2	ecstasy.mp. or exp 3, 4 methylenedioxymethamphetamine/	3673
3	cannabis.mp. or exp cannabis/	21349
4	cocaine.mp. or cocaine/	42593
5	methamphetamine.mp. or exp methamphetamine/	13394
6	amphetamine/ or amphetamine.mp.	29176
7	crystal meth*.mp.	420
8	exp illicit drug/ or illicit drug*.mp.	10826
9	illicit substance*.mp.	1673
10	exp street drug/ or street drug*.mp.	11333
11	exp child/ or exp adolescent/ or Adolescen*.mp. or exp adolescence/	3018279
12	teenage*.mp.	20901
13	young people.mp.	26123
14	youth.mp. or juvenile/	69351
15	exp young adult/ or emerging adult.mp.	799670
16	exp college student/ or college student*.mp.	20064
17	neuropsychological test.mp. or exp neuropsychological test/	175230
18	cognitive neuroscience/ or neuroscience.mp. or exp neuroscience/	45195
19	exp neuroimaging/ or neuroimag*.mp.	201591
20	brain imag*.mp.	15387
21	structural imag*.mp.	1953

22	exp nuclear magnetic resonance imaging/ or functional imag*.mp. or exp positron emission tomography	67001
23	exp spectroscopy/ or spectroscop*.mp.	756323
24	magnetic resonance imag*.mp.	502275
25	exp functional magnetic resonance imaging/ or functional magnetic resonance imag*.mp.	446378
26	structural magnetic resonance imag*.mp.	2323
27	Magnetic resonance spectroscopy.mp.	160309
28	exp electroencephalogram/ or electroencephalogra*.mp. or exp electroencephalography/	165549
29	exp electrocorticography/ or electrocorticogra*.mp.	4005
30	exp diffusion tensor imaging/ or Diffusion Tensor Imag*.mp.	16017
31	exp magnetoencephalography/ or magnetoencephalogram*.mp.	7900
32	exp near infrared spectroscopy/ or near-infrared spectroscopy.mp	17320
33	exp working memory/ or verbal working memory.mp. or exp verbal memory/	23603
34	episodic memory*.mp.	8274
35	visuospatial working memory.mp.	618
36	verbal fluency test*.mp.	859
37	exp executive function/ or executive function*.mp.	30937
38	1 or 2 or 3 or 4 or 5 or 6 or 7 or 8 or 9 or 10	109291
39	11 or 12 or 13 or 14 or 15 or 16	3407709
40	cognition.mp.	195385
41	17 or 18 or 19 or 20 or 21 or 22 or 23 or 24 or 25 or 26 or 27 or 28 or 29 or 30 or 31 or 32 or 33 or 34 or 35 or 36 or 37 or 40	1898479
42	38 and 39 and 41	2485

43	limit 42 to (english language and humans and yr="1990 -Current" and (case reports or clinical study or clinical trial or comparative study or controlled clinical trial or dataset or journal article or observational study or randomized controlled trial)	2127
